# Formation and Decay of the Dehydrogenated Parent Anion upon Electron Attachment to Dialanine

**DOI:** 10.1002/chem.201102433

**Published:** 2012-02-28

**Authors:** David Gschliesser, Violaine Vizcaino, Michael Probst, Paul Scheier, Stephan Denifl

**Affiliations:** a Institut für Ionenphysik und Angewandte Physik and Center for Molecular Biosciences Innsbruck, Leopold-Franzens-Universität Innsbruck, Technikerstrasse 256020 Innsbruck (Austria)

**Keywords:** gas-phase reactions, mass spectrometry, metastable compounds, molecular dynamics, peptides

## Abstract

**Abstract:**

The dehydrogenated parent anion [*M*−H]^−^ is one of the most dominant anions formed in dissociative electron attachment to various small biomolecules like nucleobases and single amino acids. In the present study, we investigate the [*M*−H]^−^ channel for the dipeptide dialanine by utilizing an electron monochromator and a two-sector-field mass spectrometer. At electron energies below 2 eV, the measured high-resolution ion-efficiency curve has a different shape to that for the single amino acid alanine, which is explained by the altered threshold energies for formation of [*M*−H]^−^ determined in quantum chemical calculations. Moreover, the structure of the formed [*M*−H]^−^ anion is further studied by investigating the unimolecular and collision-induced decay of this anion. Trajectory calculations have been carried out to aid the interpretation of the experimentally observed fragmentation patterns.

## Introduction

The electron-attachment process to biomolecules has gained considerable interest since it was discovered that low-energy electrons can induce single- and double-strand breaks in DNA.[1,2] In such a reaction, a free electron with a specific kinetic energy attaches resonantly to a molecule leading to a temporary negative-ion state (TNI). Once formed, different relaxation processes of the TNI are available:[3] 1) radiative stabilization, 2) spontaneous emission of the quasi-bound electron (autodetachment), and 3) molecular dissociation. Radiative stabilization, like that recently proposed for dicyanoacetylene,[4] is a rather scarce process, whereas autodetachment and dissociation are much faster and thus more likely under isolated conditions. The lifetime of the TNI may be considerably enhanced for macromolecules and solvated molecules, in which efficient intra- or intermolecular redistribution of the energy deposited by the electron may be possible.[5] Low-energy electrons are generated in abundance by the interaction of ionizing radiation with biological tissue.[1,2] Hence free-electron capture processes need to be thoroughly understood for modeling and predicting damage by ionizing radiation. Subsequently, also electron scattering and attachment[6–8] on DNA building blocks was studied experimentally and theoretically in the gas phase as well as in the condensed phase. Such investigations on electron attachment were also extended to amino acids,[9–19] which are the building blocks of peptides and proteins. DNA is packed and arranged around the latter (histones). Hence, electron-scattering processes with amino acids are also relevant for modeling radiation damage. For small biomolecules like nucleobases, amino acids, and so forth, and other small organic molecules, attachment of a low-energy electron with a typical kinetic energy of a few electron volts leads often dominantly to the closed-shell dehydrogenated molecular anion [*M*−H]^−^ [Eq. (1)]:

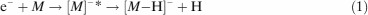

in which [*M*]^−^* describes the TNI formed by the resonant electron capture. Above the threshold of electronic excitation, the decay pattern may change in favor of smaller fragment anions formed by multiple bond ruptures in the molecules. Although a variety of single amino acids has already been studied, free-electron attachment to peptides in the gas phase has been investigated to a much lesser extent.[20–24] Collision-type experiments with peptides using ions,[25,26] photons,[27] or neutral particles[28,29] have also been carried out previously. Negative-ion mass spectra recorded at two different electron energies were reported in reference [20] for the dipeptide dialanine and some polypeptides of alanine, whereas in references [23,24, anion-efficiency curves as a function of the electron energy were shown for dialanine. These studies showed that [*M*−H]^−^ is also one of the major anions formed in dissociative electron attachment to small peptides.

**Figure 1:**
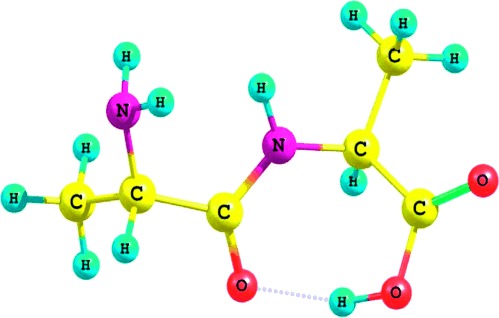
Molecular structure of dialanine.

All previous electron-attachment measurements were carried out with standard ion sources, that is, the electron-energy resolution was >0.5 eV. A measurement with better electron-energy resolution, which would allow vibrational structures in the [*M*−H]^−^ ion yield (if formed at all) to be resolved, to the best of our knowledge, has not been carried out yet. Such structures turned out to be characteristic for single amino acids.[13–15,17,30] In the present study we utilized a hemispherical electron monochromator providing an electron resolution of about 120 meV and determined the [*M*−H]^−^ ion yield as a function of the electron energy for the dipeptide dialanine (see Figure 1] for the chemical structure). Since for dialanine more sites are available for hydrogen loss than for single amino acids, we have carried out quantum chemical calculations to investigate the site of the hydrogen loss. Moreover, by utilizing a two-sector-field mass spectrometer we investigated metastable and collision-induced decays of [*M*−H]^−^ at different resonance energies to shed some light on the isomeric structure of the [*M*−H]^−^ formed. Trajectory calculations for the anions that had undergone hydrogen loss were carried out to understand the experimental observations.

## Results and Discussion

**Figure 2:**
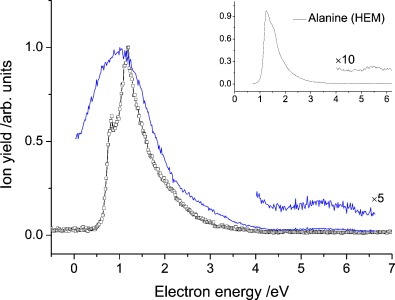
Anion efficiency curve of the dehydrogenated parent anion [*M*−H]^−^ for dialanine measured with the high-resolution electron monochromator (HEM; electron energy spread about 120 meV), two-sector-field mass spectrometer[23] (2SF-MS; electron energy spread about 1 eV), and in the inset for alanine[30] measured with the HEM. □=Dialanine (HEM); —=Dialanine (2SF-MS).

Figure 2 shows the anion-efficiency curve of the dehydrogenated parent anion [*M*−H]^−^ for dialanine measured with a hemispherical electron monochromator and a two-sector-field instrument. The high-energy resolution data shows a main resonance at about 1.17 eV and a further peak on the low-energy side that is centered at 0.81 eV. For comparison, the measurement with the two-sector-field instrument yields only one broad peak in this energy region due to the much lower energy resolution of about 1 eV.[23] Due to the about three orders of magnitude higher electron current, this instrument has a higher sensitivity at the expense of resolution of any structural features. A peak at higher electron energies close to 5.5 eV is clearly visible in the sector-field data, which is discussed in detail below. First we will focus on the ion yield at low electron energies.

**Low-energy ion yield at approximately 1 eV**: In addition to the ion yield for dialanine, [*M*−H]^−^ for the single amino acid alanine[30,31] is also included in Figure 2. A pronounced change of the low-energy peak structure close to 1 eV can be observed when going from the single amino acid unit to the dipeptide. [*M*−H]^−^ formed upon dissociative electron attachment (DEA) to alanine shows at electron energies below 3 eV an asymmetric peak structure with the steep onset starting at about 0.8 eV, a maximum at about 1.25 eV, and at least one shoulder at about 1.52 eV on the high-energy side. Other aliphatic amino acids like valine,[32] proline,[33] and (iso)leucine[34] show a very similar shape for [*M*−H]^−^ at low energies. Lowering the electron-energy-beam resolution to about 50 meV led to the same principal shape[35] and a step-type structure with dips at 1.24, 1.36, and 1.62 eV for alanine could be clearly resolved. In reference [35] these dips were interpreted as cusps due to the competition between DEA and vibrational excitation of ν(OH) up to *ν*=4. For molecules with a carboxyl group (starting from simple organic acids[36] like, for example, formic acid (HCOOH) up to amino acids) it was concluded that the low-energy structures exclusively result from hydrogen loss from the carboxyl group. This was explained by energetical reasons since the calculated thermodynamic threshold for the other [*M*−H]^−^ isomers was substantially higher. Indeed this prediction was also experimentally confirmed by experiments with partially methylated amino acids.[37,38]

Few theoretical studies on the origin of the peak structure and the involved orbitals have been carried out previously. Rescigno et al. predicted an attachment process with initial electron capture into the π*(CπO) orbital with subsequent vibronic coupling to the σ*(O=H) orbital,[39,40] which was previously also proposed in several electron-attachment studies (e.g., Refs. [13,17]). However, Gallup et al. later concluded on the basis of *R*-matrix calculations and experimental results that the σ*(O—H) resonance is solely involved in the dissociative electron-attachment process leading to [*M*−H]^−^.[41,42] The latter resonance model was very recently also successfully applied to nucleobases.[43] Concerning the type of resonance formed, vibrational Feshbach resonances (VFRs) were previously favored in reference [44] for glycine. Such VFRs were proposed to form through initial formation of dipole-bound anions. Although the minimum structure of glycine does not have a sufficiently high dipole moment (above the critical value of 2.5 D),[44] it was shown that other conformer isomers formed by the thermal heating process when vaporizing the sample do. We also calculated the dipole moment of various dialanine conformers and the three energetically lowest structures possess a dipole moment above 4 D, that is, above the critical value.[23] However, it should be noted that a sufficiently large polarizability can also lead to VFRs as shown in reference [42.

This leads to the question as to whether the features observed here for dialanine at 0.81 and 1.17 eV can be assigned like in the case of amino acids to a vibrational structure? We calculated the thermodynamic thresholds for dialanine for the various isomers of [*M*−H]^−^. These thresholds have been computed by using G4(MP2).[45] This quantum chemical extrapolation method is optimized for the calculation of thermochemical properties. The reaction thresholds are obtained as the difference between the ground-state energies of the products and reactants. The corresponding derived values are listed in Table 1] and have an estimated accuracy of ±0.1 eV. At low electron energies close to 1 eV two isomers (hydrogen loss from a carboxyl or amide group) are energetically accessible with similar thresholds. This is in striking contrast to single amino acids in which hydrogen loss from the carboxyl group is the only available channel at these energies. All other thresholds listed in Table 1] are at least about 0.5 eV higher, that is, these isomers will not contribute to the peaks at 0.81 and 1.17 eV. Therefore the [*M*−H]^−^ ion yield may a priori be assigned to a vibrational structure of the amide ν(NH) stretch or the ν(OH) stretch, which have very similar frequencies. We ruled out the latter stretching mode because although the calculated thresholds tend to be in general higher than the experimental ones, the calculated threshold for the loss of hydrogen from the carboxyl group (H_cbx_) does not match the first peak at 0.81. However, based on the results discussed below, we favor an alternative explanation for the peak structure in that a site-selective bond-cleavage process leading to different [*M*−H]^−^ isomers at the two resonances takes place. Hence, we assign the first feature at 0.81 eV to the isomer with loss of hydrogen from the amide group (H_amide_) and the 1.17 eV peak to the isomer with loss of H_cbx_.

**Table 1:** Calculated threshold energies for different isomers of the dehydrogenated parent anion of dialanine. The corresponding hydrogen-loss sites are italicized in the linear formulas.

H loss site	Anion isomer	Threshold [eV]
carboxyl group	NH_2_CH(CH_3_)CONHCH(CH_3_)CO*O*^−^	0.91
amide group	NH_2_CH(CH_3_)CO*N*CH(CH_3_)COOH^−^	0.80
amino group	*N*HCH(CH_3_)CONHCH(CH_3_)COOH^−^	2.13
	carbon site	NH_2_CH(CH_3_)CONH*C*(CH_3_)COOH^−^	2.13
		NH_2_CH(*C*H_2_)CONHCH(CH_3_)COOH^−^	2.43
NH_2_*C*(CH_3_)CONHCH(CH_3_)COOH^−^		1.60
NH_2_CH(CH_3_)CONHCH(*C*H_2_)COOH^−^	1.56

From the high-resolution measurements one cannot reach a definite conclusion as to whether the peak structure is formed by two different isomers or from the isomer with loss of H_amide_. To solve this question we utilized the two-sector-field mass spectrometer and studied the fragmentation pattern of the anion [*M*−H]^−^ formed at low electron energies. Such studies utilizing the mass-analyzed ion kinetic energy (MIKE) scan technique (see the Experimental Section) provide insight into the composition of [*M*−H]^−^. Unfortunately, the two-sector-field mass spectrometer has an energy resolution of about 1 eV, that is, both resonances (0.81 and 1.17 eV) will contribute unresolved to the ion yield. It turned out that at the electron energy close to 1 eV, at which [*M*−H]^−^ is formed most efficiently (see Figure 2), the MIKE scan does not show any metastable decay of [*M*−H]^−^. This may be ascribed to the low excess energy (equal to the initial electron energy minus the threshold energy for the reaction channel for an endothermic reaction like the present one) of the dehydrogenated anion formed at this energy. Thus one can assume that a relaxed [*M*−H]^−^ anion exits the ion source and does not decay spontaneously on the way to the detector.

**Figure 3:**
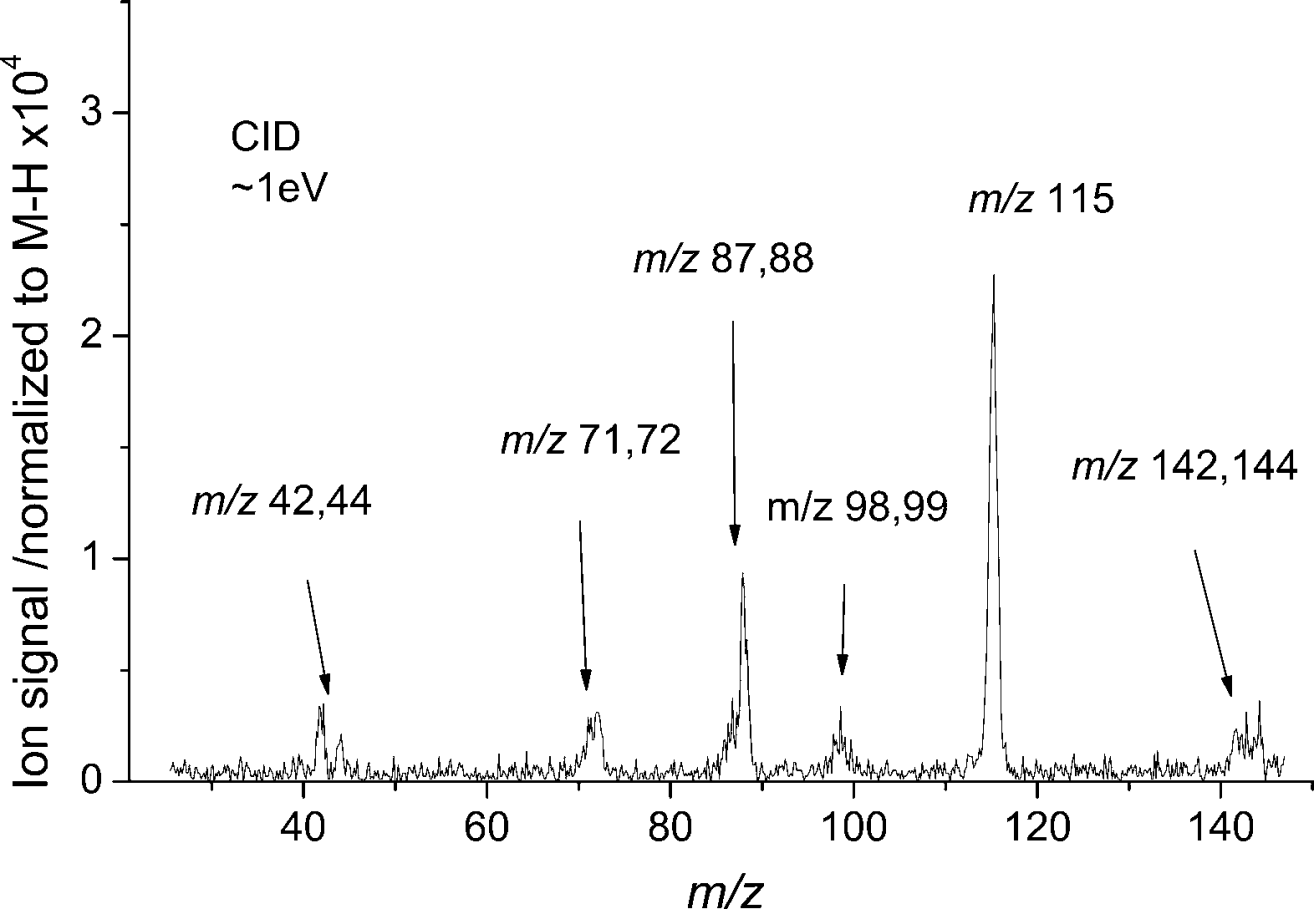
MIKE scan of the collision-induced decay (CID) of [*M*−H]^−^ for dialanine formed at the initial electron energy of about 1 eV.

Thus, only the collision-induced dissociation (CID) of [*M*−H]^−^ (*m*/*z* 159) between the magnetic and electric sectors may give insight about such structural isomerism. Therefore we carried out investigations on CID and the corresponding spectrum for the 1 eV resonance of [*M*−H]^−^ is shown in Figure 3. The CID spectrum shows a rich pattern with fragment anion peaks at *m*/*z* 42, 44, 71, 72, 87, 88, 98, 99, 115, 142, and 144 with the highest ion signal for *m*/*z* 88 and *m*/*z* 115. Dissociation after collision-induced activation can be understood as a statistical reaction, in which after initial electronic excitation the excess energy is randomly distributed over the vibrational degrees of freedom. An indication of such a process can also be found in the Gaussian shape of the decay peaks in the MIKE spectra, that is, the kinetic-energy release follows a Maxwell–Boltzmann distribution.[38

**Figure 4:**
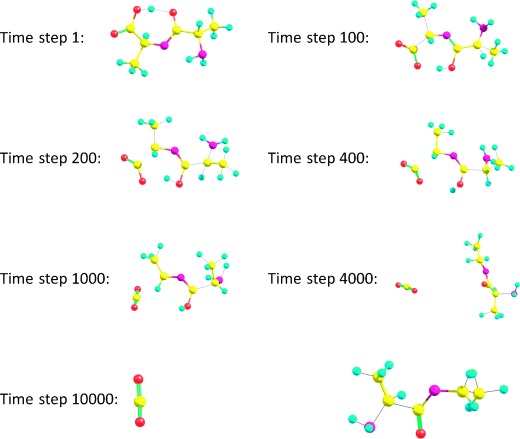
Trajectory for the loss of CO_2_ starting from [*M*−H]^−^ dialanine isomer without hydrogen at the amide group (see text).

We note that several peaks are observed in the CID spectrum, in which an instantaneous assignment of a peak to a certain fragment anion is not straightforward. Hence we carried out trajectory calculations for [*M*−H]^−^ without H_cbx_, H_amide_, hydrogen from an amino group (H_amin_), and hydrogen from two carbon sites (the carbon atom close to an amide group, H_C-amide_, and that close to an amino group, H_C-amin_), respectively. The technical details are described in the section below. General observations were that the lowest-energy set of trajectories showed no dissociations but only several hydrogen transfers. Trajectories leading to fragmentation are listed in Table 2] for the different isomers. In the simulations three trajectories led to peptide bond cleavage. For example, the hydrogen from the carboxyl group was removed initially, but subsequently a proton was transferred from the amino group leading to the supposed [NHCH(CH_3_)COOH]^−^. The simulations frequently showed such proton-transfer reactions, which in many cases led to stabilizations of the [*M*−H]^−^ anions. No trajectory without initial removal of H_amin_ led to dissociation. The most abundant fragment anion in CID is observed at *m*/*z* 115. The simulations elucidate two possible reaction pathways leading to this anion: 1) initial hydrogen loss from the amide site, subsequent proton transfer from the carboxyl group (two trajectories), and CO_2_ removal or 2) loss of CO_2_ after hydrogen loss from the carboxyl group (one trajectory). The former trajectory is shown as an example in Figure 4] and a movie of the dynamics can be found in the Supporting Information.

**Table 2:** Fragment anions, their mass, and the corresponding neutral fragments obtained in trajectory calculations for different [*M*−H]^−^ isomers (*m*/*z* 159) for dialanine (see text). Whether these masses were observed in the experimental CID spectrum at approximately 1 eV and approximately 5.5 eV, respectively, is also indicated.

Initial site of H loss	Fragment anions	Mass	Corresponding neutral fragment(s)	CID 1 eV	CID 5.5 eV
H_cbx_	NH_2_CHCONHCH(CH_3_)COO^−^	144	CH_3_	×	×
	NH_2_CH(CH_3_)CONHCH(CH_3_)^−^	115	CO_2_	×	
	NHCH(CH_3_)COOH^−^	88	NHCHCO+CH_3_	×	×
	CH(CH_3_)COO^−^	72	NH_2_CHCH_3_+HNCO	×	
	NHCH(CH_3_)CO^−^	71	H+NHCH(CH_3_)+CO_2_	×	
H_amide_	NH_2_CH(CH_3_)COHNCH(CH_3_)^−^	115	CO_2_	×	×
	CH(CH_3_)COHNCH(CH_3_)^−^	99	NH_2_+CO_2_	×	
H_C-amide_	NHCH(CH_3_)CONHCH(CH_3_)COOH^−^	141	H_2_O		×
H_C-amin_	NH_2_CH(CH_3_)COO^−^	88	NH_2_C(CH_3_)CO		×

If we compare the fragments observed in the trajectory calculations with the CID spectrum at 1 eV, it turns out that all fragments seen in the calculations for the [*M*−H]^−^ isomer without H_amide_ and H_cbx_ are also observed in CID (see Table 2) and the most abundant are *m*/*z* 88 and *m*/*z* 115. Experimentally, as well as in the simulation, we assume a statistical decay after the excess energy is distributed in the vibrational degrees of freedom in the isomer. From the comparison it follows that both isomers are formed at about 1 eV. We also note that we can observe more fragments in the CID than in the simulation. These are the weakly abundant ions at *m*/*z* 42, 44, 87, 98, and 142. The absence of these channels in the simulations may be explained by the limited number of trajectories run, as well as the relatively short timescales (see the trajectory calculations in the Experimental Section), which may obscure slow, sequential decay reactions. However, the present findings show that at the electron energy close to 1 eV a new fragmentation channel is available that is not present for amino acids. Such a remarkable site-selective fragmentation process in biomolecules within a relatively narrow energy range was previously observed in nucleobases.[8,46

**The resonance at approximately 5.5 eV**: For the resonance at approximately 5.5 eV all dissociation channels are energetically open. The electron energy is already a few electron volts above the thresholds for the different isomers and thus a considerable amount of excess energy is deposited into [*M*−H]^−^. This leads to the formation of a number of smaller fragment anions formed promptly in the ion source (see reference [23] for an overview of all detectable fragments formed). The resonance is very weakly present in the measurement using the electron monochromator setup, which may be a consequence of the approximately 10 times longer period until anions reach the detector.[47] By utilizing the two-sector-field mass spectrometer we can obtain unimolecular decays of [*M*−H]^−^ extending to the microsecond regime.

**Figure 5:**
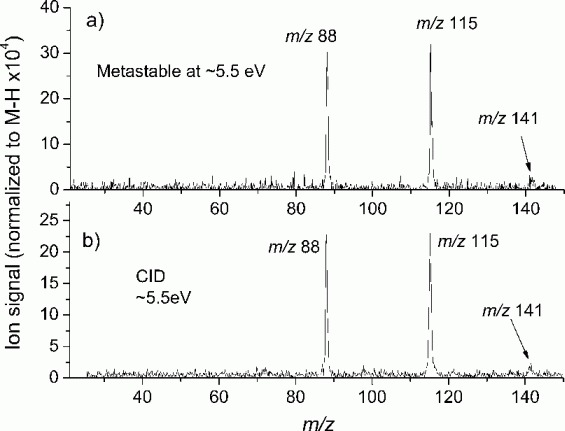
MIKE scan of the a) metastable decay and b) collision-induced decay (CID) of [*M*−H]^−^ for dialanine formed at the electron energy of 5.5 eV.

Figure 5a shows the corresponding MIKE scan of the unimolecular decay reactions of [*M*−H]^−^, *m*/*z* 159, where three decay channels are present. Two dominant peaks at mass-to-charge ratios (*m*/*z*) of 88 and 115 can be observed. These metastable decay channels have also been recently reported in reference [24; here we detect a further (weak) decay into *m*/*z* 141, that is, likely the loss of H_2_O from [*M*−H]^−^. In previous electron-attachment studies with dialanine the anion at *m*/*z* 88 was also observed abundantly at electron energies between 5 and 6 eV,[20,23,24] as a prompt dissociation product formed in the ion source. On the basis of exact mass peak determination, the chemical composition of this anion was assigned to [NHCH(CH_3_)COOH]^−^ in reference [20. It can result from the cleavage of the peptide bond OC—NH between the two alanine moieties upon direct electron capture into the σ*(C—N) orbital.[22] Our present results show that we also form this anion through a two-step process with initial hydrogen loss. The other decay products observed in the metastable time regime can be ascribed to [*M*−H−CO_2_]^−[20]^ at *m*/*z* 115 and [*M*−H−H_2_O]^−^ at *m*/*z* 141, respectively. We also measured the electron energy dependence of the metastable decays of [*M*−H]^−^ into *m*/*z* 88 and *m*/*z* 115, respectively (not shown). In agreement with reference [24] the decays only occur above 2.5 eV, peaking at 5.5 eV (and further at 8.5 eV), and have an almost identical shape.

We also recorded a CID spectrum of [*M*−H]^−^ formed at the electron energy of approximately 5.5 eV. The resulting spectrum is shown in Figure 5b and resembles the spectrum observed for the metastable decay at this electron energy and shows only the same three peaks at *m*/*z* 88, 115, and 141. At this point the question arises as to why we do not observe in the CID at 5.5 eV a rich fragmentation pattern like at low electron energies. In general, one possible explanation may be that different isomers of [*M*−H]^−^ are formed at the two different electron energies. In this case the trajectory calculation would again shed light on the isomers involved. For example, the presence of the isomer with loss of H_carboxyl_ and H_amide_ could be excluded, since otherwise the CID at approximately 5.5 eV would show the fragments listed in Table 2] for these isomers. Instead, the simulation indicates that isomers with initial hydrogen loss from carbon sites may contribute to the CID peaks at *m*/*z* 141 and *m*/*z* 88 (see Table 2). These isomers are not formed at approximately 1 eV for energetic reasons. However, the main CID peak at *m*/*z* 115 is not observed in the trajectory calculations for these isomers.

Thus another explanation based on the consideration of the excess energy of the [*M*−H]^−^ anion may describe better the decay of [*M*−H]^−^. An electron with approximately 5.5 eV kinetic energy brings a considerable amount of excess energy into [*M*−H]^−^ that likely leads to electronic excitation and/or subsequent rearrangement. Moreover, the captured electron may also excite only certain vibrational modes. In any case, the [*M*−H]^−^ anion may enter the collision already in a highly activated state, which leads to fragmentation before equilibration of the energy deposited by the collision with nitrogen takes place. This prior activation may then lead to the dominance of specific fragmentation channels. As a consequence we probe at 5.5 eV the collision-induced decay of a nonrelaxed anion. In this case the trajectory calculations may give limited information for the analysis of the experimental CID spectrum. We note that analogous molecular dynamics (MD) simulation for the decay of [*M*−H]^−^ for the single amino acid valine showed only one weaker fragmentation channel (COOH^−^) but not the main fragmentation channel.[38] Nevertheless different decay peaks formed at the 1 and 5.5 eV resonances, observed in both the metastable as well as collision-induced decays, showed previously the different nature of the dehydrogenated valine anion. On the contrary, here we find that the dominant fragmentation channels observed in CID at approximately 1 eV and approximately 5.5 eV are the same.

## Conclusion

We have investigated the formation and decay of the dehydrogenated parent anion formed through electron attachment to the dipeptide dialanine. High-resolution measurements combined with quantum chemical calculations of the threshold energies of different isomers indicate that the [*M*−H]^−^ isomers without the hydrogen from the carboxyl group and the amide group are formed in the low-energy region. We confirmed this hypothesis by experimental collision-induced decay spectra and MD simulations. This formation of two different isomers is in marked contrast to single amino acids, in which exclusive hydrogen loss from the carboxyl group is possible at low electron energies due to energetic reasons. In contrast to the [*M*−H]^−^ anion at approximately 1 eV, the [*M*−H]^−^ anion formed at an electron energy of about 5.5 eV is highly unstable upon fragmentation and represents an intermediate product of sequential decays.

## Experimental Section

The high-resolution electron-attachment experiment was performed with a crossed-beam setup in combination with a quadrupole mass spectrometer (for more details see reference [31]). A commercially available dialanine sample from Sigma–Aldrich (stated purity ≥99 %) was placed in an oven and heated to temperatures of about 413 K. The oven was attached to a copper capillary with a 1 mm opening that ended close to the collision chamber of the electron monochromator. This region was the crossing zone of the monochromatized electron beam and the neutral effusive beam of molecules. Anions formed were extracted by a weak electric field into a quadrupole mass spectrometer. The mass-selected anions were finally detected by a channel electron multiplier that was operated in single-pulse counting mode. The electron-energy spread was about 120 meV in the measurement (at an electron current of about 20 nA). The energy spread was determined with the well-known s-wave attachment reaction leading to Cl^−^ from CCl_4_. This resonance at about 0 eV was also used for the calibration of the energy scale.

Metastable (unimolecular) and collision-induced decays of the dehydrogenated dialanine anion [*M*−H]^−^ were studied with a two-sector-field instrument[48,49] of reversed Nier–Johnson magnetic sector–electric sector configuration. The ion source of the mass spectrometer was a standard Nier-type ion source in which an effusive beam of dialanine molecules, evaporated in an oven (heating temperature typically 398 K), was crossed with an electron beam of about 1 eV resolution. Anions formed were accelerated by 8 kV and subsequently the mass and energy were analyzed. After passing the electric sector, anions were detected with a channel electron multiplier operated in single-pulse counting mode. The electron beam current used was 10 μA with an energy spread of about 1 eV. Decay reactions of [*M*−H]^−^ in the field-free region between the magnetic and electric sector were monitored by MIKE scans.[50] MIKE scans were recorded by first setting the magnet to select a certain “parent” anion with mass *m*_0_ (in the present study [*M*−H]^−^) and then scanning the electric-sector field voltage *U*. Whereas a non-decaying anion with mass *m*_0_ passes the electric sector at *U*_0_, a fragment anion with mass *m*_1_ formed by a decay of the precursor anion with *m*_0_ only passes the electric sector at the reduced sector field voltage [Eq. (2)]:

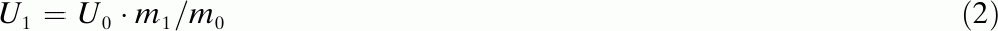


Metastable (unimolecular) decomposition pathways of [*M*−H]^−^ in the time window between about 10 and 30 μs were studied. Collision-induced decays of [*M*−H]^−^ were also investigated using the MIKE scan method. However, in this case a gas cell between the magnetic and electric sector was filled with stagnant nitrogen gas inducing decays through collisions.

**Trajectory calculations**: We tried to obtain qualitative insight into the fragmentation process of the [*M*−H]^−^ anions by performing a limited set of trajectory calculations. In one set of calculations [*M*−H] was described quantum chemically by the B3LYP[51] density functional and the 6-31G*[52] basis set, whereas in the other one the PW91[53] density functional with the same basis set was used. Both functionals are of the same overall quality but represent different concepts, B3LYP being more empirical and PW91 more rigorously founded. The dynamics was generated by the atom-centered density matrix propagation (ADMP) method.[54,55] The program Gaussian 09[56] was used for all calculations. The dissociation reaction was then simulated in the following way: 1) The anionic species with the H missing on the amino, amide, or carboxyl group was geometrically relaxed. 2) Boltzmann-distributed velocities were assigned to the atoms. The individual velocity components were created by appropriate scaling of random numbers. 3) This process was repeated and five trajectories each for kinetic energies of 7, 14, and 20 eV were calculated for all isomers (and for each of the two density functionals). 4) Each trajectory was propagated for 2000 time steps of 0.1 fs each. It should be noted that five trajectories of 400 fs each do not allow for good statistics. It must be kept in mind, though, that the computational expense of such ab initio molecular dynamics calculations is still very high, even with the modest 6-31G* basis set. Both density functionals behaved similarly, with PW91 leading to more dissociations at 14 eV total kinetic energy.
